# Bronchial artery embolization for haemothorax and haemoptysis caused by primary lung cancer

**DOI:** 10.1002/rcr2.529

**Published:** 2020-02-05

**Authors:** Shota Yamamoto, Shunsuke Kamei, Yusuke Kondo, Shinichiro Hiraiwa, Terumitsu Hasebe, Fumio Sakamaki

**Affiliations:** ^1^ Department of Radiology Tokai University Hachioji Hospital, Tokai University School of Medicine Tokyo Japan; ^2^ Department of Respiratory Medicine Tokai University Hachioji Hospital, Tokai University School of Medicine Tokyo Japan; ^3^ Department of Pathology Tokai University Hachioji Hospital, Tokai University School of Medicine Tokyo Japan

**Keywords:** Bronchial artery embolization, haemoptysis, haemothorax, hypoxaemia, lung cancer

## Abstract

Primary lung cancer (PLC) presents with various symptoms. However, there have been no reports of PLC causing haemothorax and haemoptysis simultaneously. We present an unusual case of massive haemothorax and haemoptysis caused by a PLC, in which haemostasis was secured with interventional radiology. A 58‐year‐old woman was hospitalized for a right secondary pneumothorax associated with emphysema. Chest computed tomography showed a mass shadow at the right lower lobe and on the right parietal pleura. Three days after air drainage, about 2000 mL of bloody pleural effusion accompanied by massive haemoptysis was observed. Haemoglobin concentration decreased to 4.9 g/dL and the patient was treated with selective embolization of the bronchial artery and the intercostal arteries. A diagnosis of PLC was made based on pleural fluid cytology. The patient was transferred to the palliative care hospital three months later without recurrence of haemothorax and haemoptysis.

## Graphical abstract

This is the first report of primary lung cancer causing a massive haemothorax and haemoptysis simultaneously. We believe it is also the first such case in which haemostasis was secured using bronchial artery embolization.

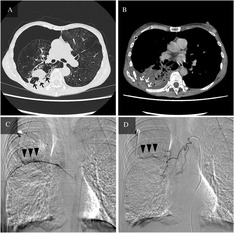

## Introduction

Primary lung cancer (PLC) presents with various symptoms. Haemoptysis is common and is reported by 9–64% of patients diagnosed with PLC 1. However, massive haemothorax originating from PLC is extremely rare 2, and there have been no reports of PLC causing haemothorax and haemoptysis simultaneously. Here, we report an unusual case of massive haemothorax and haemoptysis caused by PLC, which was treated with selective embolization of the bronchial artery and the intercostal arteries in order to achieve haemostasis. To the best of our knowledge, this is the first report of PLC causing a massive haemothorax and haemoptysis simultaneously. We believe it is also the first such case in which haemostasis was secured using bronchial artery embolization (BAE).

## Case Report

**Figure 1:**
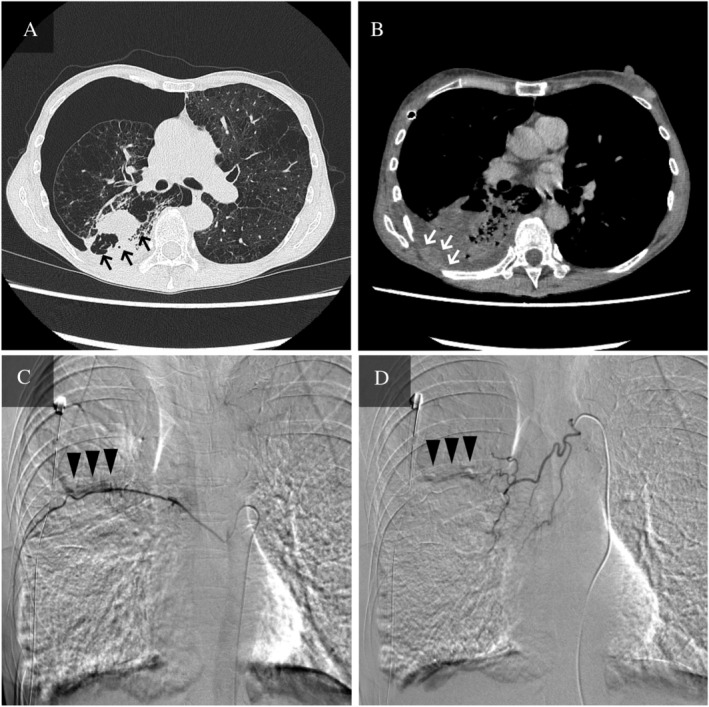
Chest computed tomography (CT) and angiographic findings. (A) CT on admission day revealed a right secondary pneumothorax of emphysema. There was a pleura‐attached mass shadow with cavity at the periphery of the right lower lobe (S6); the diameter was 35 mm (black arrows). There was partial thickness of the parietal pleura and no pleural effusion. (B) CT with contrast agent on the day of haemothorax and haemoptysis. There was consolidation in the right middle/lower lobes, and a right pleural effusion had appeared. There was an 18‐mm‐diameter nodule invading the chest wall at the right eighth rib level (white arrows). A chest tube was inserted via the front chest. (C) Angiography of the right eighth intercostal artery showed contrast agent in the vicinity of the pleural nodule (black arrowheads). This site was considered the origin of bleeding. (D) Angiography of the right bronchial artery revealed contrast agent in the same area as the right eighth intercostal artery (black arrowheads).

**Figure 2:**
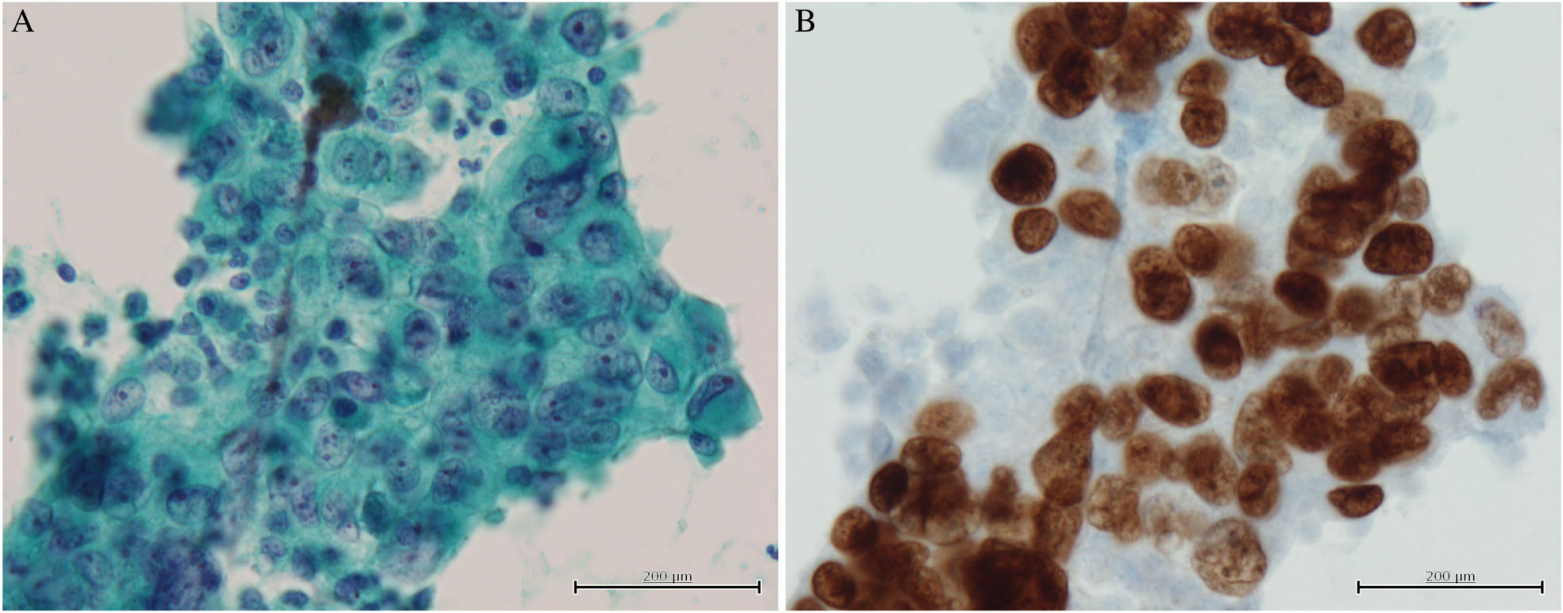
Cytological findings of pleural fluid. (A) Tumour cells forming epithelioid clusters. Most of the tumour cells had pale cytoplasm and hyperchromatic nuclei with prominent nucleoli (Papanicolaou's stain, 400× magnification). (B) The nuclei of atypical cells were scored positive for thyroid transcription factor‐1 (TTF‐1) following immunocytochemistry (immunostain, 400× magnification).

A 58‐year‐old woman was admitted to our hospital with right secondary pneumothorax associated with emphysema. She had a smoking history of 30 packs‐per‐year, but no history of chest trauma. On physical examination, we observed a tympanic sound was heard over the right chest. Her heart rate was 87 beats/min and blood pressure 106/50 mmHg. Laboratory data included a white blood count of 14,300/ mm^3^, haemoglobin 9.5 g/dL, platelets 688,000/ mm^3^, C‐reactive protein (CRP) 0.94 mg/dL, and carcinoembryonic antigen (CEA) of 7.5 ng/dL. Plain chest computed tomography (CT) showed a right pneumothorax, which was caused by severe emphysema. There was a pleura‐attached mass shadow at the periphery of the right lower lobe, partial thickness of the parietal pleura, and no pleural effusion on the day of admission. The thyroid had no masses (Fig. 1A). Three days after a chest tube was inserted for air drainage, 1685 mL of bloody pleural effusion accompanied by massive haemoptysis was observed. The patient's blood pressure decreased to 90/58 mmHg and her haemoglobin concentration had fallen to 4.9 g/dL. The patient received 4 U of packed red blood cells. A subsequent CT with contrast agents demonstrated consolidation in the right middle/lower lobes and the nodule invading the chest wall at the right eighth rib level (Fig. 1B). Due to the continuous bleeding, we performed emergency BAE using a co‐axial system consisting of a 1.7‐Fr microcatheter and 4‐Fr shepherd hook/cobra catheter. No remarkable extravasation of contrast agent was noted, but arteriography at both the right eighth intercostal artery and the right bronchial artery showed contrast in the vicinity of the pleural nodule (Fig. 1C, D). Therefore, we embolized the arteries feeding the tumour using a gelatin sponge particle. The day after BAE, the amount of blood‐tinged and serous chest tube drainage gradually began to decrease. The tube was removed 12 days after BAE. We did not execute the pleurodesis, as we were concerned that it might lead to a new lung injury. This is because there was a possibility that talc or OK‐432 could reach the parenchyma through the pulmonary fissure of the pneumothorax. Pleural fluid cytological examination yielded a diagnosis of poorly differentiated carcinoma. The tumour cells had clear cytoplasm and hyperchromatic nuclei with prominent nucleoli (Papanicolaou's stain) (Fig. 2A) and were expressed for thyroid transcription factor‐1 (TTF‐1) (Fig. 2B). Whole body CT did not find any extra‐thoracic lesions or thyroid tumours. Therefore, we made a diagnosis of PLC, clinically staged as T3N0M1a (stage IVA). The patient did not want to receive any chemotherapy and was transferred to the palliative care hospital three months after BAE without recurrence of haemothorax and haemoptysis.

## Discussion

Massive haemoptysis is one of the most challenging conditions to treat. Conservative management of massive haemoptysis carries more than 50% risk of mortality 3. BAE is an established procedure for the management of haemoptysis. For patients with haemoptysis resulting from PLC, a recent study showed that BAE was an effective treatment modality with a favourable safety profile. The technical and clinical success rates of BAE in patients with PLC are reportedly 77–100% and 58.3–89%, respectively 4.

Massive haemothorax can be caused by pulmonary angiosarcoma, mesothelioma, and lung metastasis of hepatocellular carcinoma. However, massive haemothorax originating from PLC is extremely rare 2. There have been no reports of PLC causing haemothorax and haemoptysis simultaneously. According to the angiographic findings, the right bronchial artery (intra‐pulmonary vessel) and the intercostal artery (extra‐pulmonary vessel) fed the same tumour. Here, we suggest that the primary lesion had invaded the chest wall and was fed by both arteries. Microvascular rupture might be induced by tumour necrosis or the mechanical stress of pneumothorax, and therefore patients may present with dual symptoms. Kamiyoshihara et al. reported one case of PLC causing a massive haemothorax in which haemostasis was secured with BAE 5. They suggested that thoracotomy would impose a heavy burden on patients, and therefore opted to carry out BAE, which is a less invasive procedure. There were three reasons why we chose the gelatin sponge for haemostasis. First, there are some cases in which permanent embolization could induce late tumour necrosis, which could lead to fatal bleeding. Second, and in contrast with temporary embolic materials, there have been no reports that permanent embolic materials significantly reduce the rebleeding rate associated with haemoptysis caused by PLC. Late recurrences are typically due to disease progression 4. Finally, the gelatin sponge provides the option of re‐intervention when haemoptysis recurs 3.

We conclude that PLC can be the origin of massive haemothorax and haemoptysis, particularly when PLC develops towards the pleura. Furthermore, we suggest that BAE is useful for controlling the bleeding from a PLC in critical situations.

### Disclosure Statement

Appropriate written informed consent was obtained for publication of this case report and accompanying images.
